# A cross-sectional study on antibiotic prescription in a teaching hospital in Ghana

**DOI:** 10.11604/pamj.2020.35.12.18324

**Published:** 2020-01-15

**Authors:** Pilar Garcia-Vello, Fareeda Brobbey, Bruno Gonzalez-Zorn, Courage Kosi Setsoafia Saba

**Affiliations:** 1 Projects of Molecular Biology and Biotechnology, Faculty of Pharmacy, Complutense University of Madrid, Madrid, Spain; 2 Specialist Pharmacy, Tamale Teaching Hospital, Tamale, Ghana; 3 Animal Health Department, Faculty of Veterinary, Complutense University of Madrid, Madrid, Spain; 4 Biotechnology Department, Faculty of Agriculture, University for Development Studies, Tamale, Ghana

**Keywords:** Use of antibiotics, public health, Tamale Teaching Hospital, Ghana

## Abstract

**Introduction:**

Antibiotic misuse is the paramount factor for antibiotic resistance. Tamale Teaching Hospital (TTH), located in Ghana’s Northern Region, is the biggest tertiary hospital in the Northern half of the country and consequently one of the biggest prescribers of antibiotics. Understanding the use of antibiotics in the TTH and providing information that could be inferred to develop strategies for antibiotic prescription is of extreme importance in this era of multiple and pan-resistant strains of pathogenic microorganisms.

**Methods:**

A cross-sectional study on the use of antibiotics at TTH in the Northern region of Ghana was performed. Data were collected by reviewing 10% of patients’ files from January to June 2015 and then assessed for its appropriateness against the criteria based on the British National Formulary (BNF) 2015 and BNF children 2013-2014. Results were expressed in frequencies and percentages.

**Results:**

A total of 617 patients’ records were included in this study. Up to 385 cases of different antibiotic misuse were found, comprising of 335 errors in prescriptions and 50 non-completed treatments. The most common prescription error was made on treatment duration (29.6%). The potential interactions were 16.7%.

**Conclusion:**

The study revealed a high burden of antibiotics misuse in TTH. This suggests a need for the development of an antibiotic stewardship programme for the hospital.

## Introduction

Access to medication, particularly to antibiotics, is growing all over the world paving the way for unprecedented medical and societal developments as far as life expectancy is concerned. Nevertheless, the recent increases in antibiotic resistant bacteria of all genera have necessitated global and coordinated efforts to minimize its effects by controlling the use of antibiotics and prompting the scientific community to perform routine surveillance of microbial populations to determine the extent of the resistance [1]. The causes of antibiotic resistance are complex and their consequences affect both humans and animals worldwide [2]. Antibiotic misuse is the paramount driver of resistance, which ranges from a combination of overuse in many parts of the world; misuse and lack of access to appropriate treatment in others and failure to complete treatment courses. Besides, inherent microbial characteristics and human behaviour at different levels of society also contribute to the spread of antibiotic resistance due to substantial increases in the availability and ease of movement within and between countries [3]. Ghana is a West African country that provides a universal health care system called the National Health Insurance Scheme (NHIS) [4], which is designated for residents in Ghana. Though it varies widely within the country, it ensures that most of the hospitals, clinics and pharmacies are stocked with basic drugs that are used to treat patients to ensure access to primary health care [4,5]. There have been several studies on antibiotics resistance in Ghana [6-8], which comprise of distinct facets of antibiotic resistance research. However, considering the large population size of Ghana and the life expectancy of 62/64 years at birth [9] as well as more than 200 hospitals [4], those studies seem to be excessively focused on the most developed areas of the country, particularly Kumasi and Accra. Tamale Teaching Hospital (TTH) is located in Tamale, in Ghana’s Northern Region. TTH was chosen for this study because it is a referral hospital for the northern half of the country and some parts of Burkina Faso, Togo and Ivory Coast. About 20 000 patients are admitted to the hospital annually, with an approximate mortality rate of 5% (20 004 patients in 2013 and 1009 died) [10], making the hospital one of the biggest caregivers of the area and consequently one of the biggest prescribers of antibiotics. The general objective was to assess the use of antibiotics in TTH and provide information that could be inferred to develop an antibiotic stewardship programme in the hospital. Specific objectives were: 1) to evaluate the proportion of patients who suffered from improper antibiotic therapy; 2) to analyze the diverse improper uses of antibiotics at various units (wards), types of errors and particular kinds of antibiotics misused within the hospital; 3) to investigate the causes of improper use of antibiotics to help the hospital to curb the misuse of antibiotics.

## Methods

This was a retrospective cross-sectional study conducted in the Tamale Teaching Hospital (TTH) in Tamale, Northern Ghana. We collected 10% of patients’ available records from January-June 2015. The patients’ inclusion criteria were based on patients with a diagnosis of a disease that required antibiotics treatment or patients with any kind of antibiotic prescription. The records were handwritten medical folders stored at the hospital. A minimum sample of 50 patients’ records per ward with an antibiotic prescription was randomly obtained. Wards such as ear nose and throat (ENT), neurology and urology had fewer inpatients, so all the available folders were analysed. Data were collected with structured questionnaires by a team of pharmacists working in the hospital. Nurses and medical doctors on-duty were consulted for clarifications in cases where there were difficulties in interpreting the information. Information regarding the demographics (age, sex, weight, therapeutic procedure inpatient/outpatient, history number, date of admission, and date of discharge) and clinical data (diagnosed disease, prescriber, antibiotics prescribed, route of administration, dose, frequency and duration prescribed and completion of the prescription) were identified and documented. Consecutively, the collected data were analysed for its appropriateness using the criteria based on the British National Formulary 2015 (BNF) [11] and BNF children 2013-2014 [11] guidelines. It was considered an antibiotic misuse or error any discrepancy between these guidelines and the prescription given at the hospital or the lack of compliance to a correct prescription.

**Quality assessment of the use of antibiotics:** 1) correspondence between diagnosis and prescription; 2) doses (amount administered), frequency (number of times administered daily) and duration (how many days) of the treatment; 3) potential interaction between dissimilar prescribed antibiotics (other medical or nutritional potential interactions were not considered) in cases were prescribed antibiotics could produce a non-desired alteration of functions; 4) treatment compliance: if administered or stopped as prescribed. Patients that were consequently discharged, on oral antibiotics, were noted as completed treatments; in lieu of a follow up with patients. Incomplete treatments due to decease were not tallied. Data were then coded and entered into Excel^®^ for analysis. The results were expressed in frequencies and percentages.

## Results

**Baseline characteristics:** a total of 617 patients’ records from Tamale Teaching Hospital (TTH) were analysed, from seven different wards. The information sourced from inpatients’ records is presented in Table 1.

**Table 1:** Baseline characteristics (n = 617)

Ward	Children^b^	ENT^b^	Female^b^	Male^b^	Neurol^b^	Surgical^b^	Urology^b^	Total
Number of folders	104	46	126	137	54	111	39	617
Men	52 (50)	17 (37)	0 (0.0)	137 (100)	48 (88.9)	84 (75.7)	35 (89.7)	373(60.5)
Women	52 (50)	28 (60.9)	126 (100)	0 (0.0)	6 (2.24)	27 (24.3)	4 (10.3)	243(39.4)
Unclear sex	0 (0.0)	1 (2.2)	0 (0.0)	0 (0.0)	0 (0.0)	0 (0.0)	0 (0.0)	1(0.2)
Nº Discharged	91 (87.5)	43 (93.5)	94 (74.6)	109(79.6)	45 (83.3)	95 (85.6)	34	511(82.8)
Nº Dead	13 (12.5)	3 (6.5)	32 (25.4)	28 (20.4)	9 (16.7)	16 (14.4)	5	106(17.2)
Prescriptions made by physician assistants	0 (0.0)	0 (0.0)	6 (4.8)	12 (8.8)	0 (0.0)	1 (0.9)	0 (0.0)	19(3.1)
Patients on non-antibiotic treatment	16 (15.4)	10 (21.7)	30 (23.8)	29 (21.2)	8 (14.8)	8 (7.2)	0 (0.0)	101(16.4)

a Table values are n (column %) for categorical variables.

b Numbers may not sum to total due to missing data, and percentages may not sum to 100% due to rounding.

**Antibiotics analysis:** a variety of antibiotics were prescribed in TTH. Thirty-seven different antibiotics were found in patient records. Intravenous administration was designated as IV. The most prescribed were Metronidazole IV (36.5% of patients), Ceftriaxone IV (35.3%), Ciprofloxacin IV (24.5%), Metronidazole oral (24.6%), Cefuroxime oral (18.6%), Ciprofloxacin oral (17.7%), Co-amoxiclav oral (15.6%), Co-amoxiclav IV (13.3%), Cefuroxime IV (12%), Azithromycin oral (7.9%) and Gentamicin IV (4.4%). Every ward had a unique prescription pattern. The most prescribed antibiotics were Ceftriaxone IV in Paediatrics (51.0%), Neurology (57.4%) and Urology (46.2%); followed by Metronidazole IV in Surgical (73.0%), Male ward (37.2%) and Female ward (29.4%) and Co-amoxiclav IV in ENT (34.8%). We found 385 different cases of antibiotic misuse in the 617 folders, 335 in prescriptions and 50 non-compliments of treatments. The most common prescription error found was the duration of treatments (29.6%), close to 97% of them were treatments that did not cover the minimum of days required and 3% were excessively long treatments cases (Table 2).

**Table 2:** Errors analysis

Errors in prescription counted by antibiotic mistaken (n=335)^c^								
Type of error	Pediatrics^b^	ENT^b^	Female^b^	Male^b^	Neurology^b^	Surgical^b^	Urology^b^	Total^b^
**Diag-pres^d^**	27 (24.5)	1 (12.5)	17 (29.3)	29 (37.7)	0 (0.0)	8 (15.1)	0 (0.0)	82 (24.5)
**Doses**	27 (24.5)	0 (0.0)	4 (6.9)	4 (5.2)	2 (15.4)	6 (11.3)	1 (6.3)	44 (13.1)
**Doses per day**	5 (4.5)	2 (25.0)	6 (10.3)	1 (1.3)	2 (15.4)	3 (5.7)	9 (56.3)	28 (8.4)
**Duration**	29 (26.4)	3 (37.5)	21 (36.2)	31 (40.3)	3 (23.1)	10 (18.9)	2 (12.5)	99 (29.6)
**Erratic**	6 (5.5)	0 (0.0)	6 (10.3)	4 (5.2)	1 (7.7)	9 (17.0)	0 (0.0)	26 (7.8)
**Interaction**	16 (14.5)	2 (25.0)	4 (6.9)	8 (10.4)	5 (38.5)	17 (32.1)	4 (25.0)	56 (16.7)
**Total errors**	110 (100)	8 (100)	58 (100)	77 (100)	13 (100)	53 (100)	16 (100)	335(100)
**Errors in prescription counted by** Patients affected **(n=617)**								
**Alteration**	**Pediatrics^b^**	**ENT^b^**	**Female^b^**	**Male^b^**	**Neurology^b^**	**Surgical^b^**	**Urology^b^**	**Total^b^**
**Total folders with errors**	51 (49)	9 (19.6)	49 (38.9)	47 (34.3)	11 (20.4)	34 (30.6)	12 (30.8)	213 (34.5)
**Discharged patients with errors^e^**	46 (50.6)	8 (18.6)	39 (41.5)	47(43)	10 (22.2)	25 (26.1)	9 (26.5)	184 (36.0) [n=511]
**Dead patient with errors^f^**	5 (38.5)	1 (33.3)	10 (31.2)	11(39.3)	1 (11.1)	5 (31.2)	3 (60)	36 (34.9) [n=106]

a Table values are n (%) for categorical variables.

b Numbers may not sum to total due to missing data, and percentages may not sum to 100% due to rounding.

c Numbers are calculated summing every error, multiple errors can correspond to one folder or to one antibiotic.

d No correspondence diagnoses prescription

e Percentage calculated upon discharged patients

f Percentage calculated upon dead patients

g Percentage calculated upon no medical prescriptions

* Not adequate prescription for the patient condition according to the guidelines cited in the methodology.

It is also notable to say that, a physician assistant perpetrated only one of the cases presented in Table 2. Two of the errors recorded due to the non-correspondence diagnoses-treatment were because of the lack of an antibiotic for a disease that required it. Potential interactions between antibiotics were vigorously examined. Potential interactions represented 16.7% of the prescription errors. The most common potential interaction observed was the co-administration of Ceftriaxone and Ciprofloxacin (35.7% of the potential interactions), also some frequent potential interactions were the combination of Cefuroxime with Ciprofloxacin (8.9%), Cefuroxime with Co-amoxiclav (7.1%) and Ceftriaxone with Amoxicillin (5.4%). Non-compliance of treatments by the nursing team was mostly because of the absence of doses (36%), especially in the surgical ward, where it accounted for nearly 60% of the non-compliance errors. Additional leading causes of non-compliance were: the cessation of medication without justification (32%) and providing different frequency than prescribed (18%) (Table 3).

**Table 3:** Non-compliance of treatments by nursery team

Type of error	Pediatrics^b^	ENT^b^	Male^b^	Neurology^b^	Surgical^b^	Urology^b^	Female^b^	Total^b^
**Concurrently^c^**	1 (33.3)	0 (0.0)	0 (0.0)	0 (0.0)	0 (0.0)	0 (0.0)	0 (0.0)	1 (2.0)
**Frequency^d^**	1(33.3)	0 (0.0)	1(12.5)	0 (0.0)	2 (11.8)	0 (0.0)	5 (27.7)	9 (18.0)
**Doses Absence^e^**	1(33.3)	0 (0.0)	1(12.5)	0 (0.0)	10 (58.8)	0 (0.0)	6 (33.3)	18 (36.0)
**Late start^f^**	0 (0.0)	0 (0.0)	2 (25.0)	0 (0.0)	0 (0.0)	0 (0.0)	0 (0.0)	2 (4.0)
**Cessation^g^**	0 (0.0)	0 (0.0)	4 (50.0)	4 (100)	1 (5.9)	0 (0.0)	7 (38.9)	16 (32.0)
**Not given^h^**	0 (0.0)	0 (0.0)	0 (0.0)	0 (0.0)	0 (0.0)	0 (0.0)	0 (0.0)	0 (0.0)
**Not prescribed^i^**	0 (0.0)	0 (0.0)	0 (0.0)	0 (0.0)	4 (23.5)	0 (0.0)	0 (0.0)	4 (8.0)
**Total**	3	0	8	4	17	0	18	50 (100)

a Table values are n (%) for categorical variables.

b Numbers are calculated summing every error, multiple errors can correspond to one folder or to one antibiotic.

c Concurrently administration of new and old medication after a prescription change.

d Administration with a frequency different than prescribed.

e Antibiotic not administered continuously because of the skip of some doses

f The antibiotic was not administered the prescribed day without reported justification

g The administration of the antibiotic stopped without reported justification

h The antibiotic was not administered without reported justification

i Administration of a not prescribed antibiotic without reported justification

**Errors per antibiotic:** in addition, errors were analyzed according to the antibiotic. Metronidazole IV accounted for the highest errors in TTH (21.5% of all antibiotics’ errors); approximately 50% of the errors committed in metronidazole IV administration were due to the short duration of treatments prescribed. It was followed by Ceftriaxone IV (14.3% of the total errors). Ceftriaxone IV commonest error was also because of the short duration of treatments (31.6% of ceftriaxone errors) and its prescription without a disease that required it (31.6%). Ciprofloxacin IV (12.5% of all antibiotics errors) was commonly prescribed without a need (33.3% of the ciprofloxacin errors). Besides, Ciprofloxacin IV was commonly prescribed in low doses without evidence of kidney disease, representing 30.3% of the Ciprofloxacin errors. Cefuroxime IV (11.3%) presented a common error: the mix-up in the strength of Cefuroxime between its IV and oral formulations. Intravenous formulation is commonly given twice daily when it should be given thrice daily. Oral form strength is also ill prescribed, administered thrice daily when it should be given twice daily. Other antibiotics with high error rates were Cefuroxime oral (5.6% of all antibiotic errors), Co-amoxiclav IV (5.3%), Co-amoxiclav oral (4.9%) and Azithromycin oral (3.8%).

## Discussion

To the best of our knowledge, this study is one of the first in-depth investigations on the use of antibiotics in the Tamale Teaching Hospital. There is paucity of data in the prescription of antibiotics in the Northern region of Ghana and generally in the country. This study may serve as reference literature to ensure proper prescription of antibiotics not only in the Teaching Hospital but Ghana as a whole. The results suggest a high proportion of antibiotic misuse. This outcome corresponds with other studies conducted in different African regions [12-16]. It is also remarkable the high prevalence of non-completion of treatments, off-label prescriptions, potential interactions, and inadequate protocols implementation. The prescription also fell short of WHO guidelines for antibiotic prescription [17, 18]. The plausible reason for such findings is the absence of a well-structured antibiotic stewardship programme and the lack of consciousness on antibiotics management among health-care professionals [18]. The high frequency of wrong administration of Cefuroxime is also alarming. The analysis showed that this error is due to a mix-up in the frequency between its IV and oral formulations. Similar maladministration has been found in different African hospitals [19].

The general practice, however, is the administration of partial treatments and the lack of follow-ups. This is not an acceptable practice. Different studies indicated the financial burden as one of the reasons for defaulting [20-23]. Where economical barriers exist, solidarity is encouraged among patients from the same ward to share one or two doses of different medications. Those doses are administered and never recorded. Both patients and hospital workers must be educated on the futility that one single dose of antibiotics represents and the need to use antibiotics judiciously to curb the increasing resistance of bacteria to antibiotics. Cases of errors made by physician assistants were uncommon; by that virtue, they did not pose a serious problem concerning patients’ safety. However, the high proportion of prescriptions that were not made by doctors in some wards showed inadequate supervision by pharmacists and medical doctors. This was also reported by another study in the country [24]. We observed that the absence is especially high on night admissions when at least one medical doctor is supposed to be available to supervise diagnosis and prescriptions. In addition, poor quality of records was encountered. Most history folders were uncompleted and in bad condition. Furthermore, pharmaceutical and laboratory records were external papers attached to patients’ folders that were easily lost. Having all the information in the same folder will help patients’ follow-up during their hospital stay or future admissions. It will also help the record of hospital drug shortages and the pharmaceutical interventions performed [25].

**Study limitations:** the search was restricted to only 617 patients’ records out of more than 6000 from seven wards. Also, our analysis was based exclusively on patients’ records hence we had access only to the information available in the records without recourse to the missing pages. Consequently, the folder may not necessarily provide a complete picture of the patients’ time in the hospital. Laboratory tests and patients’ history of the use of antibiotics could not be considered because of the misplacement of registration numbers and folders by patients and hospital workers, duplicate registration of patients, the attendance to different hospitals and the easy accessibility of antibiotics without a medical prescription in Ghana. These factors made it difficult to monitor patients’ records to provide more precise data on the prescription of antibiotics to patients.

## Conclusion

The study revealed a high burden of antibiotics misuse in the Tamale Teaching Hospital. This suggests an urgent need for an antibiotic stewardship programme in the hospital that must be strictly followed. There is also a need for a more conscious sensitization programme on the use of antibiotics and resistance among healthcare workers in the hospital.

### What is known about this topic

- Antibiotic resistance is one of the biggest threats to health and security nowadays;
- Antibiotic resistance occurs naturally, as a survival adaptation of the bacteria; nevertheless, antibiotics misuse in humans and animals accelerates the process;
- It is crucial to improve the prescription of antibiotics and the management in hospitals to minimize the apparition of antibiotic-resistant bacteria.

### What this study adds

- The results suggest a high proportion of antibiotic misuse. High prevalence of non-completion of treatments, off-label prescriptions, potential interactions and inadequate protocols implementation;
- The most common errors were the prescription of Ciprofloxacin IV without need, more frequency administration of Cefuroxime and the co-administration of Ceftriaxone and Ciprofloxacin;
- The administration of partial treatments, lack of follow-ups and poor quality of records is a common undesirable practice.
